# The *Salt Flip*: Sensory mitigation of salt (and sodium) reduction with monosodium glutamate (MSG) in “Better‐for‐You” foods

**DOI:** 10.1111/1750-3841.15354

**Published:** 2020-08-10

**Authors:** Jeremia Halim, Ali Bouzari, Dan Felder, Jean‐Xavier Guinard

**Affiliations:** ^1^ Department of Food Science and Technology University of California Davis California USA; ^2^ Pilot R&D Berkeley California USA

**Keywords:** better‐for‐you foods, consumer testing, monosodium glutamate (MSG), preference mapping, salt and sodium reduction, Salt Flip, sensory quality

## Abstract

**Abstract:**

We tested the hypothesis that reduced‐salt versions of four “better‐for‐you” dishes enhanced with monosodium glutamate (MSG) through a “Salt Flip” in an amount that still substantially reduced total sodium matched the consumer acceptance of normal‐salt versions. Three versions each—standard recipe with normal salt, reduced salt, and reduced salt with MSG, of four dishes—roasted vegetables (RV), quinoa bowl (QB), savory yogurt dip (SD), and pork cauliflower fried rice (CR) were evaluated by 163 consumers for overall liking and liking of appearance, flavor, and texture/mouthfeel on the nine‐point hedonic scale, preference, adequacy of flavor, saltiness, and aftertaste on just‐about‐right (JAR) scales, likeliness to order, and sensory characteristics by check‐all‐that‐apply. For each dish, the MSG recipe was liked the same (or significantly more for SD, *P* < 0.05) than the standard recipe, and better than the reduced salt recipe for QB and CR. The same was true of likeliness to order. MSG recipes of QB and SD were significantly preferred to the standard recipes, with no difference for RV and CR. MSG recipes were consistently described as “delicious,” “flavorful,” and “balanced.” Penalty‐lift analysis showed that “delicious,” “flavorful,” “balanced,” “fresh,” and “savory”; and “bland,” “rancid,” and “bitter,” were positive and negative drivers of liking, respectively. Two of three uncovered preference clusters, accounting for 68% of consumers, consistently liked MSG recipes, and the same or more so than standard recipes. We conclude that MSG can successfully be used to mitigate salt and sodium reduction without compromising consumer acceptance of better‐for‐you foods.

**Practical Application:**

The Salt Flip offers a promising dietary sodium reduction strategy through the addition of monosodium glutamate (MSG) to reduced‐salt, savory, better‐for‐you foods that does not compromise consumer acceptance of their sensory profile.

## 1 INTRODUCTION

Sodium intake is an important public concern in most countries, as it is linked to several chronic diseases such as hypertension, cardiovascular disease, renal failure, and osteoporosis, among others. Reducing sodium intake has been clinically shown to lower blood pressure short term (Sacks et al., 2001) and mortality from stroke and heart disease long term (Law, 2000). The main dietary source of sodium is sodium chloride (NaCl) or table salt. A significant challenge to the public health community and the food industry alike is that salt reduction typically compromises the palatability of foods (Beauchamp, Bertino, & Moran, 1982; Breslin & Beauchamp, 1997). Flavor‐enhancing ingredients such as monosodium glutamate (MSG) thus offer a possible sensory strategy for the mitigation of salt reduction with regard to palatability. Recently, the use of MSG has been acknowledged by the National Academic of Sciences, Engineering, and Medicine as a viable strategy to reduce sodium in the food supply (Stallings et al., 2019).

MSG is the sodium salt of L‐glutamic acid, the most abundant amino acid in nature, constituting up to 8% to 10% of most dietary proteins either as free glutamate or bound to other amino acids. The amount of sodium in MSG (12.28 g/100 g) is one third of the sodium in salt (39.34 g/100 g), which makes it a promising salt alternative in sodium reduction strategies. The majority of glutamate intake is from that naturally present in food, with only a small amount derived from MSG seasoning (Henry‐Unaeze, 2017). Ever since the discovery of glutamate's unique, umami taste by Dr. Ikeda in 1908 (Kawamura & Kare, 1987), MSG has been widely used as a flavor enhancer in savory foods. MSG imparts the umami taste via a T1R1/T1R3 heteromeric receptor. Its umami taste can be further potentiated by the 5’ ribonucleotides inosine monophosphate and guanosine monophosphate, both naturally found in foods like beef and mushrooms (Giovanni & Guinard, 2001; Zhang et al., 2008). Yamaguchi & Kimizuka (1979) also verified that some intensification of salty taste takes place when umami substances, such as MSG or other nucleotides are present. In the United States, MSG is classified by the Food and Drug Administration (FDA) as a “Generally Recognized as Safe” substance. The usage level as a food additive is 0.1% to 0.8% by weight.

Research has previously documented the successful application of MSG to substitute for salt without compromising the sensory profile or consumer acceptance of several foods and dishes—soups and broths (Altug & Demirag, 1993; Ball, Woodward, Beard, Shoobridge, & Ferrier, 2002; Chi & Chen, 1992; Chung et al., 2019; Jinap et al., 2016; Mojet, Heidema, & Christ‐Hazelhof, 2004; Okiyama & Beauchamp, 1998; Roininen, Lahteenmaki, & Tuorila, 1996; Yamaguchi & Takahashi, 1984), pork patties (Chun et al., 2014), sausages (Dos Santos, Campagnol, Morgana, & Pollonio, 2014; Woodward, Lewis, Ball, & Beard, 2003), potato chips, puffed rice (Buechler & Lee, 2019) and mixed dishes (Leong, Kasamatsu, Ong, Hoi, & Loong, 2016). Those studies support up to 30% reduction in sodium from salt with MSG to maintain taste parity. The strategy has not been tested as fully in complex dishes, however, particularly those deemed “better‐for‐you” foods. The term “Better‐for‐You Foods” was introduced recently to describe those plant‐forward foods with a desirable nutritional profile that consumers should be eating more of. Some research initiatives have focused on the class of foods and beverages labeled as such (Elliott, 2012).

Negative consumer attitudes toward MSG, as documented for United States (Wang & Adhikari, 2018) and New Zealand consumers (Prescott & Yong, 2002), have also been an obstacle to its broader adoption. Yet it is worth noting that sensory properties weighed more than ingredient information in the acceptance of vegetable soups with MSG (Prescott & Young, 2002), and that college students’ response to MSG could be improved through education (Jin & Han, 2019). With more convincing scientific evidence and better communication, those attitudes have improved and the outlook for the use of MSG in foods is positive (Mintel, 2019).

We have tested a number of sensory and culinary strategies for dietary change with some success. The most impactful was our proof‐of‐concept research on the ability of mushrooms to substitute partially for meat without compromising the flavor profile or consumer acceptance because of their umami properties (Guinard et al., 2016; Myrdal‐Miller et al., 2014). This led to the development of the (beef‐mushroom) blend and the widespread adoption of blended burgers as a healthier alternative to traditional burgers in US schools, cafeterias, and restaurants nationwide, as highlighted in *Scientific American* (Jacewicz, 2016) and Nature (2018) editorials. *Sonic's Slinger*© (Sonic Corp., Oklahoma City, OK, USA) has since come to symbolize the success of blended burgers in fast casual restaurants. We have also developed and tested, with success, other “flip” strategies for the replacement of nutritionally‐contested ingredients with healthier ones with flavor‐boosting or matching properties, that is, the Flexitarian Flip (Spencer & Guinard, 2018; Spencer, Cienfuegos, & Guinard, 2018a, 2018b) and the Dessert Flip (Kurzer, Wiriyaphanich, Cienfuegos, Spang, & Guinard, 2020).

We therefore hypothesized that because of its umami taste and flavor‐enhancing properties, MSG could be used as a flavor potentiator in reduced‐salt dishes in what essentially is a salt flip for a lesser total sodium than the normal‐salt, standard recipes without compromising the consumer acceptance of those dishes. Corollary hypotheses in this study were that reduced‐salt versions of the dishes would be liked less than the standard (regular‐salt) recipes, and that reduced‐salt recipes with MSG added would be liked more than the reduced‐salt recipes.

## 2 MATERIALS AND METHODS

### 2.1 Dishes and recipes

This research investigated the mitigation of salt and sodium reduction in four plant‐forward, better‐for‐you dishes—roasted vegetables (RV), a quinoa bowl (QB), a savory yogurt dip (SD), and pork and cauliflower fried rice (CR). The criteria for their selection were plant‐based or focused, resonant, iconic, and easy for consumers to replicate. The recipes for each dish were developed and kitchen tested at Pilot R&D (San Francisco, CA, USA) in three versions—a standard recipe with a typical salt content for the dish, a reduced‐salt recipe, and the same reduced salt recipe with MSG added at a level such that total sodium was still substantially lower than in the standard recipe. The salt (and sodium) levels for the regular or typical version of each dish were established through tastings of original iterations of the dishes at Pilot R&D. The salt (and sodium) reduction levels and the MSG addition levels were then determined in consultation with the manufacturer (Ajinomoto) for best practices in the use of their Ac'cent MSG ingredient. The sodium reduction in the reduced salt recipes, as estimated through ingredient composition, ranged from 47% to 63% for the reduced‐salt recipes and from 31% to 61% for the MSG recipes (Table 1).

**Table 1:** Sodium level (in g) for each recipe and percent sodium reduction from the standard recipe

		Added sodium level (g)		
Dish	RACC (g)	Standard recipe	Reduced salt recipe	MSG recipe
RV	110	0.27	0.14 (−47.81%)	0.19 (−31.26%)
QB	140	0.37	0.20 (−46.81%)	0.25 (−30.98%)
SD	28	0.16	0.07 (−58.56%)	0.09 (−46.46%)
CR	119	0.17	0.06 (−62.80%)	0.07 (−60.91%)

RACC, reference amount customarily consumed.

All recipes were prepared according to the specifications in Supplement A the day before the tests. On the day of the tests, the refrigerated, vacuum‐sealed, 500 g, food grade polyethylene bags with the prepared RV, QB, and CR recipes were reheated in a steam vapor oven on full (100%) steam setting to 150 °F and plated 10 to 15 min before testing time. The savory dip (SD) was kept refrigerated until session time and served at 38 °C. Serving sizes were 3 to 4 oz per plate for the RV, QB, and CR dishes, and 2 oz per cup with 5 pieces of unsalted crackers for SDs.

### 2.2 Consumers

One hundred and sixty‐three (163) consumers were recruited via advertisement and listservs from the Davis and Sacramento areas in Northern California. The screener verified consumption of both animal‐ and plant‐based ingredients such as those in the BFY foods in this study, and no food allergies. It did not inquire specifically, however, about Better‐for‐You food consumption *per se*, or MSG acceptance, so as not to reveal too much about the purpose of the research and potentially bias consumer responses. The consumers’ age ranged from 18 to 62 years, with a median of 26 years. Consumer ethnicities were 50% Asian, 28% White/Caucasian, 7% Latino, 2% Black/African, and 1% Native American.

### 2.3 Consumer test setting, design, and protocol

Testing took place in the Silverado Vineyards Sensory Theater, a central location on the UC Davis campus designed for sensory testing which can accommodate up to 50 consumers in partitioned testing stations. All samples were prepared in the adjacent Carlos Alvarez Food Innovation Laboratory.

Consumers completed two 30‐min sessions at approximately the same time on two consecutive days. Testing took place between 10 am and 6 pm. RV and then QB recipes were tested on the first day and SD and then CR recipes on the second. For each dish the order of serving of the three recipes—standard, reduced‐salt, and reduced‐salt with MSG, was randomized across consumers. Consumers were asked to cleanse their palates with water and crackers between samples; and to take a 10‐minute break between the first and the second dish on each day.

At the end of the second day, consumers completed an exit survey with questions regarding their demographics, cooking and eating habits, and attitudes regarding food and health (Supplement B).

For each recipe, consumers were asked to indicate their degree of liking overall, and then for the appearance, flavor (taste and smell), and texture/mouthfeel of the dishes on the nine‐point hedonic scale (Peryam & Pilgrim, 1957); to rate the adequacy of the flavor (taste and smell), saltiness, and aftertaste of the recipes on five‐point just‐about‐right (JAR) scales; and to describe the sensory characteristics of the recipes using a check‐all‐that‐apply (CATA) list of 16 terms—flavorful, salty, sweet, sour, bitter, complex, bland, delicious, fresh, rancid, balanced, savory, lingering aftertaste, nutty, cheesy, and meaty. Consumers were then asked how likely they would be to order the recipe at a restaurant on a five‐point bipolar scale from “I would definitely not order this dish” to “I would definitely order this dish.” They were then asked what specifically they liked and disliked about the dish in an open comment section. And finally, consumers were asked to rank the three recipes for each dish from most liked to least liked.

### 2.4 Data analysis

To compare the hedonic ratings and other parametric values of the three recipes across all consumers, two‐factor ANOVAs with a repeated measure design were conducted separately for each of the four dishes, with recipes and consumers as fixed and random effects, respectively. Duncan's multiple range test was used for *post hoc* comparisons (*P* < 0.05). We assessed the relation among the various hedonic measures with Pearson's correlation coefficient.

A nonparametric Friedman rank sum analysis was performed on the preference ranking data for each dish separately. In addition, R‐index analyses (Xia et al., 2020) were applied to these ranking data, giving an actionable choosing probability (*R*_MAT_ value) of one recipe being preferred over the others.

For each dish, penalty analyses were conducted to relate hedonic ratings to the JAR scores for flavor, saltiness, and aftertaste. The difference between the average liking score of each non‐JAR level and the average liking score of the JAR level was computed for each attribute. For each product and each attribute, an ANOVA was performed including contrast analysis in such a way that the main effect for the JAR level was zero (*α*_JAR_ = 0; Lê & Worch, 2015).

For the CATA descriptors, Cochran‐Q analyses with family‐wise controlled significance level were conducted to assess overall differences among recipes for each attribute. For multiple comparisons among the recipes, permutation tests with Benjamini–Hochberg adjusted *P*‐value (Benjamini & Hochberg, 1995) were used. This test controls for the false discovery rate and thus is more powerful and suitable for parity testing. The descriptors were also linked to the overall liking data by penalty‐lift analysis. Furthermore, correspondence analyses with chi‐square distances were conducted to examine the associations between the recipes and the CATA descriptors.

To study preference segmentation, a hierarchical clustering on principal components (HCPC) algorithm was performed on the overall liking data pooled across all dishes and recipes (12 samples in total), using Ward's criterion and the Euclidean distance. All statistical analyses were performed in R (R Core Team, Vienna, Austria).

## 3 RESULTS AND DISCUSSION

### 3.1 Hedonic ratings

The main hypothesis in this study—that reduced‐salt recipes with added MSG would be liked as much as the standard recipes, was proven correct. The reduced‐salt recipe with added MSG was liked the same as the standard recipe for RV, QB, and CR, and significantly more (*P* < 0.05) for SD (Table 2).

**Table 2:** Mean (and SEM) of overall liking, liking of appearance, flavor and texture, likeliness to order, and preference rank mean

Dish	Recipe	Overall liking	Appearance	Flavor	Texture	Likeliness to order	Mean Rank
RV	S	5.644 (0.140)^a^	4.601 (0.124)^a^	5.669 (0.140)^a^	5.804 (0.133)^a^	2.620 (0.095)^a^	2.009 (0.062)^a^
	R	5.767 (0.131)^a^	4.546 (0.121)^a^	5.773 (0.129)^a^	5.613 (0.140)^a^	2.595 (0.088)^a^	1.966 (0.058)^a^
	M	5.828 (0.138)^a^	4.577 (0.120)^a^	5.767 (0.143)^a^	5.687 (0.134)^a^	2.650 (0.092)^a^	2.024 (0.066)^a^
QB	S	5.785 (0.123)^a^	3.534 (0.131)^b^	6.147 (0.117)^a^	5.521 (0.128)^b^	2.699 (0.093)^a^	1.951 (0.064)^a^
	R	5.233 (0.127)^b^	3.933 (0.126)^a^	5.331 (0.125)^b^	5.564 (0.124)^b^	2.313 (0.086)^b^	2.294 (0.057)^b^
	M	5.908 (0.129)^a^	3.828 (0.129)^a^	6.190 (0.126)^a^	5.877 (0.119)^a^	2.847 (0.095)^a^	1.755 (0.058)^a^
SD	S	5.644 (0.139)^b^	6.012 (0.103)^a^	5.571 (0.142)^b^	6.301 (0.109)^a^	2.595 (0.102)^a^	2.144 (0.061)^b^
	R	5.810 (0.124)^ab^	6.117 (0.100)^a^	5.810 (0.119)^ab^	6.239 (0.111)^a^	2.620 (0.084)^a^	2.049 (0.059)^b^
	M	6.067 (0.123)^a^	6.135 (0.102)^a^	6.031 (0.127)^a^	6.393 (0.105)^a^	2.791 (0.095)^a^	1.807 (0.065)^a^
CR	S	4.939 (0.139)^a^	5.006 (0.131)^b^	4.951 (0.142)^a^	5.859 (0.114)^a^	2.190 (0.091)^a^	1.819 (0.060)^a^
	R	4.380 (0.135)^b^	4.945 (0.129)^b^	4.344 (0.139)^b^	5.644 (0.124)^b^	1.840 (0.075)^b^	2.371 (0.054)^b^
	M	4.933 (0.135)^a^	5.528 (0.136)^a^	4.853 (0.137)^a^	5.926 (0.116)^a^	2.178 (0.088)^a^	1.810 (0.059)^a^

S, Standard; RS, reduced salt; M, MSG recipe.

Different superscripts indicate significant differences (*P* < 0.05) among the three recipes of a particular dish for each measure.

An examination of specific hedonic ratings for appearance, flavor, and texture shows that the appearance of the MSG recipes was liked more than that of the standard recipe in the QB and CR dishes; the flavor liking ratings mirrored the overall liking ratings. And even though salt reduction and MSG addition would mostly be expected to alter the flavor of a dish, the texture of the QB with MSG was liked better than that of the other two recipes, and its appearance was liked better than that of the standard recipe (*P* < 0.05).

The corollary hypothesis that salt reduction would lower liking for the dishes was only verified for the QB and CR dishes (Table 2).

Similarly, the other corollary hypothesis that MSG addition would enhance liking of a reduced‐salt recipe was only verified for the QB and CR dishes (Table 2).

All hedonic ratings were significantly correlated with each other for all four dishes (*P* < 0.001), but we found the highest correlation between flavor liking and overall liking for all four dishes (*r* = 0.85 or higher, *P* < 0.001; Table 3).

**Table 3:** Pearson's correlation coefficients among hedonic ratings across (a) RV, (b) QB, (c) SD, and (d) CR dishes

	Overall liking	Appearance liking	Flavor liking	Texture liking	Likeliness to order
**(a) RV dishes**					
Overall liking	1.00				
Appearance liking	0.42***	1.00			
Flavor liking	0.90***	0.36***	1.00		
Texture liking	0.61***	0.36***	0.58***	1.00	
Likeliness to order	0.75***	0.39***	0.74***	0.58***	1.00
**(b) QB dishes**					
Overall liking	1.00				
Appearance liking	0.37***	1.00			
Flavor liking	0.85***	0.28***	1.00		
Texture liking	0.57***	0.39***	0.57***	1.00	
Likeliness to order	0.74***	0.38***	0.74***	0.59***	1.00
**(c) SD dishes**					
Overall liking	1.00				
Appearance liking	0.33***	1.00			
Flavor liking	0.92***	0.31***	1.00		
Texture liking	0.54***	0.54***	0.52***	1.00	
Likeliness to order	0.77***	0.28***	0.77***	0.50***	1.00
**(d) CR dishes**					
Overall liking	1.00				
Appearance liking	0.42***	1.00			
Flavor liking	0.92***	0.40***	1.00		
Texture liking	0.62***	0.53***	0.57***	1.00	
Likeliness to order	0.78***	0.38***	0.77***	0.53***	1.00

*** Significant correlation (*P* < 0.001, Bonferroni adjusted significance level for pairwise comparison at *α* = 0.001).

### 3.2 Likeliness to order

This new variable that we added to our assessment tools to account for the fact that dishes may be ordered in a restaurant setting or for takeout produced the same outcomes as the hedonic ratings of the recipes. There was no difference in likeliness to order between the standard recipes and the reduced‐salt recipes with MSG, thus verifying further our main hypothesis (Table 2). And likeliness to order was again significantly higher in the MSG recipes than in the reduced‐salt ones for QB and CR only.

### 3.3 Relation among hedonic and likeliness to order ratings

The relationship between hedonic and likeliness to order ratings was explored using Pearson's correlation coefficient across the recipes for each dish. Those with a significant correlation (*P* < 0.001, Bonferroni adjusted *P*‐value for all pairwise comparisons at family‐wise significance level of 0.001) are noted in Table 3. A highly significant correlation was observed between likeliness to order and overall liking for all four dishes (*r* = 0.74 or higher, *P* < 0.001), as well as with liking of flavor (*r* = 0.74 or higher, *P* < 0.001).

### 3.4 Preference rankings

The outcomes of the hedonic ratings on the nine‐point hedonic scale were confirmed by the preference rankings with, as expected, even greater discrimination (Table 2). Our R‐index analysis on the preference ranks found that there was no difference in preference probability between the standard recipe and the reduced‐salt recipe with added MSG for RV and CR, and that the reduced‐salt recipe with MSG actually was significantly more likely to be preferred for QB and SD (*P* < 0.05; Table 4), and the addition of MSG led to significant preference probability over the reduced‐salt recipe for QB, SD, and CR, but not RV (no preference; *P* < 0.05; Table 4).

**Table 4:** RMAT indices showing the probability of choosing the MSG recipes over the others

	Probability of choosing MSG recipe over	
Dish	Standard recipe	Reduced salt recipe
RV	49.49%	47.94%
QB	56.65%*	69.25%*
SD	61.36%*	58.40%*
CR	50.17%	70.19%*

* Significant preferences (*P* < 0.05), based on Bi and O'Mahony (2007).

### 3.5 Just‐About‐Right (JAR) ratings

Consumers’ JAR ratings of the adequacy of flavor, saltiness, and aftertaste are collapsed into three categories in Table 5—too little, just‐about‐right, or too much. For RV, the three recipes showed a similar distribution. More than 50% of consumers rated the flavor, saltiness, and aftertaste of the three recipes as JAR, with the MSG recipe receiving slightly more JAR ratings for flavor and saltiness, but not significantly. The reduced salt recipe was found to be significantly more lacking in saltiness compared to the other two recipes. For QB, more than 50% of consumers rated the reduced salt version as lacking in flavor and saltiness. In contrast, both the MSG and the standard recipe for this dish received more than 60% flavor and saltiness JAR ratings. For SD, only the MSG recipe received more than 50% JAR ratings for flavor. It also received the highest JAR selection (69.33%) for saltiness. Yet, its rating distribution was not different from that of the reduced salt recipe. Finally, for CR, the three recipes received high “too much” ratings for flavor and ‘too little’ ratings for saltiness, more than JAR ratings for each attribute. There was no difference between the JAR rating distributions of the MSG recipe and the reduced salt recipe for all three attributes.

**Table 5:** Percentage of JAR category choice by consumers across all dishes and recipes for adequacy of flavor, saltiness, and aftertaste (*n* = 163 consumers)

		Flavor				Saltiness				Aftertaste			
Dish	Recipe	TL	JAR	TM	Sig^a^	TL	JAR	TM	Sig^a^	TL	JAR	TM	Sig^a^
RV	S	10.43	54.60	34.97	a	9.82	66.87	23.31	a	9.20	59.51	31.29	a
	R	20.86	50.92	28.22	a	22.09	68.10	9.82	b	15.95	60.12	23.93	a
	M	14.72	55.83	29.45	a	14.11	76.69	9.20	a	9.20	57.67	33.13	a
QB	S	14.11	60.12	25.77	a	15.34	63.80	20.86	a	9.82	57.67	32.52	a
	R	53.37	38.04	8.59	b	62.58	34.36	3.07	b	27.61	56.44	15.95	b
	M	23.93	61.35	14.72	c	27.61	66.87	5.52	c	14.11	60.12	25.77	a
SD	S	11.04	44.17	44.79	a	13.50	56.44	30.06	a	13.50	55.83	30.67	a
	R	31.29	39.88	28.83	b	28.83	61.35	9.82	b	19.63	61.35	19.02	a
	M	23.31	50.92	25.77	b	20.25	69.33	10.43	b	16.56	55.83	27.61	a
CR	S	17.18	39.26	43.56	a	47.24	42.33	10.43	a	11.04	50.92	38.04	a
	R	33.13	21.47	45.40	b	63.80	27.61	8.59	b	18.40	47.24	34.36	a
	M	36.20	30.67	33.13	b	57.06	36.20	6.75	b	15.34	56.44	28.22	a

S, Standard; R, reduced salt; M, MSG recipe; TL, too little; JAR, just about right; TM, too much.

^a^ Different letters (a, b, c) in Sig column indicate significant differences among the JAR scores distribution, determined using CMH method and Stuart–Maxwell as post‐hoc at family wise significance level of 0.05.

**Figure 1:**
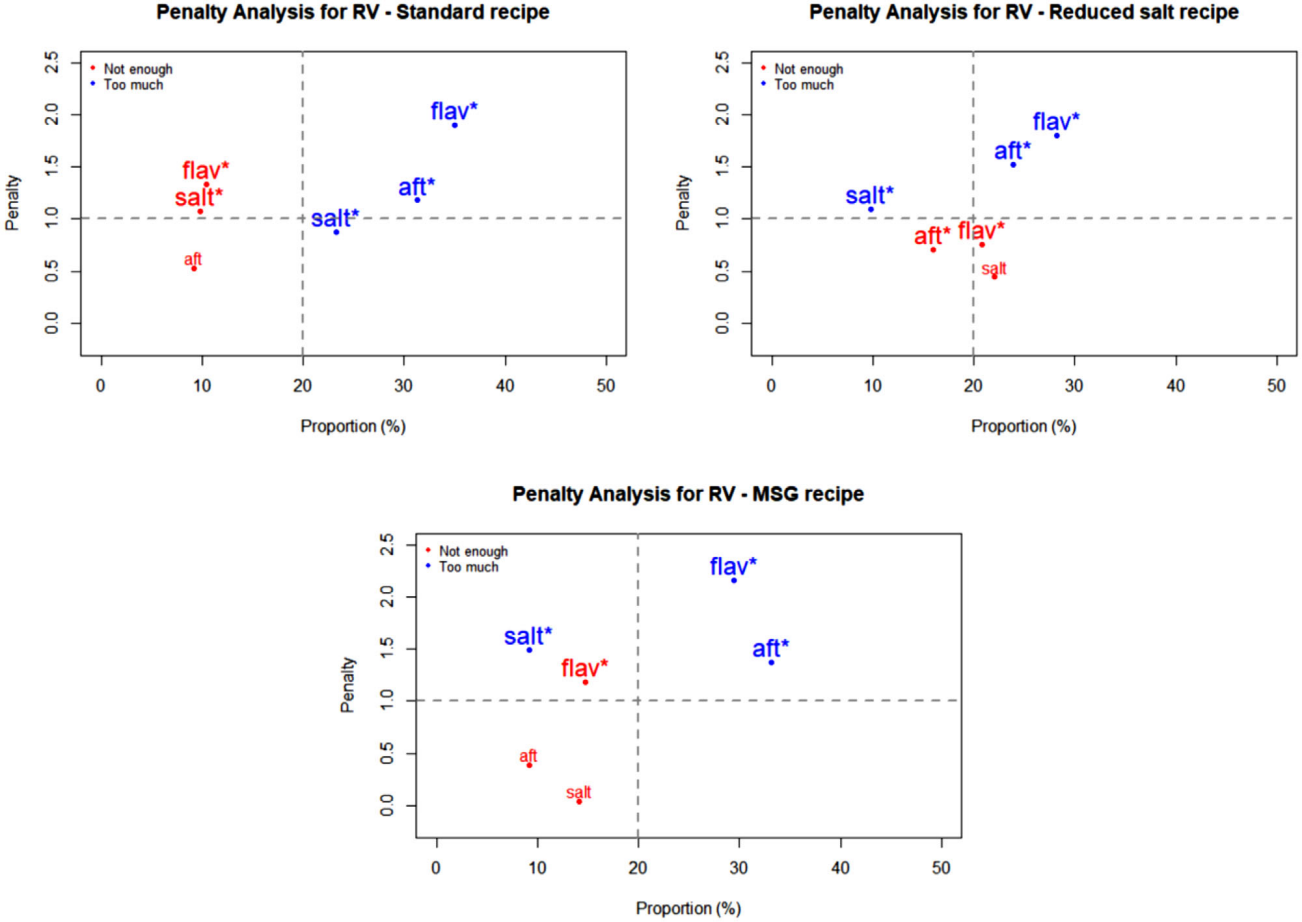
Penalty analyses of the three roasted vegetables (RV) recipes for three attributes: saltiness (“salt”), flavor (“flav”), and aftertaste (“aft”). *Note*: Significant non‐JAR categories (*P* < 0.05) are shown in larger font size and marked with “^*^.”

**Figure 2:**
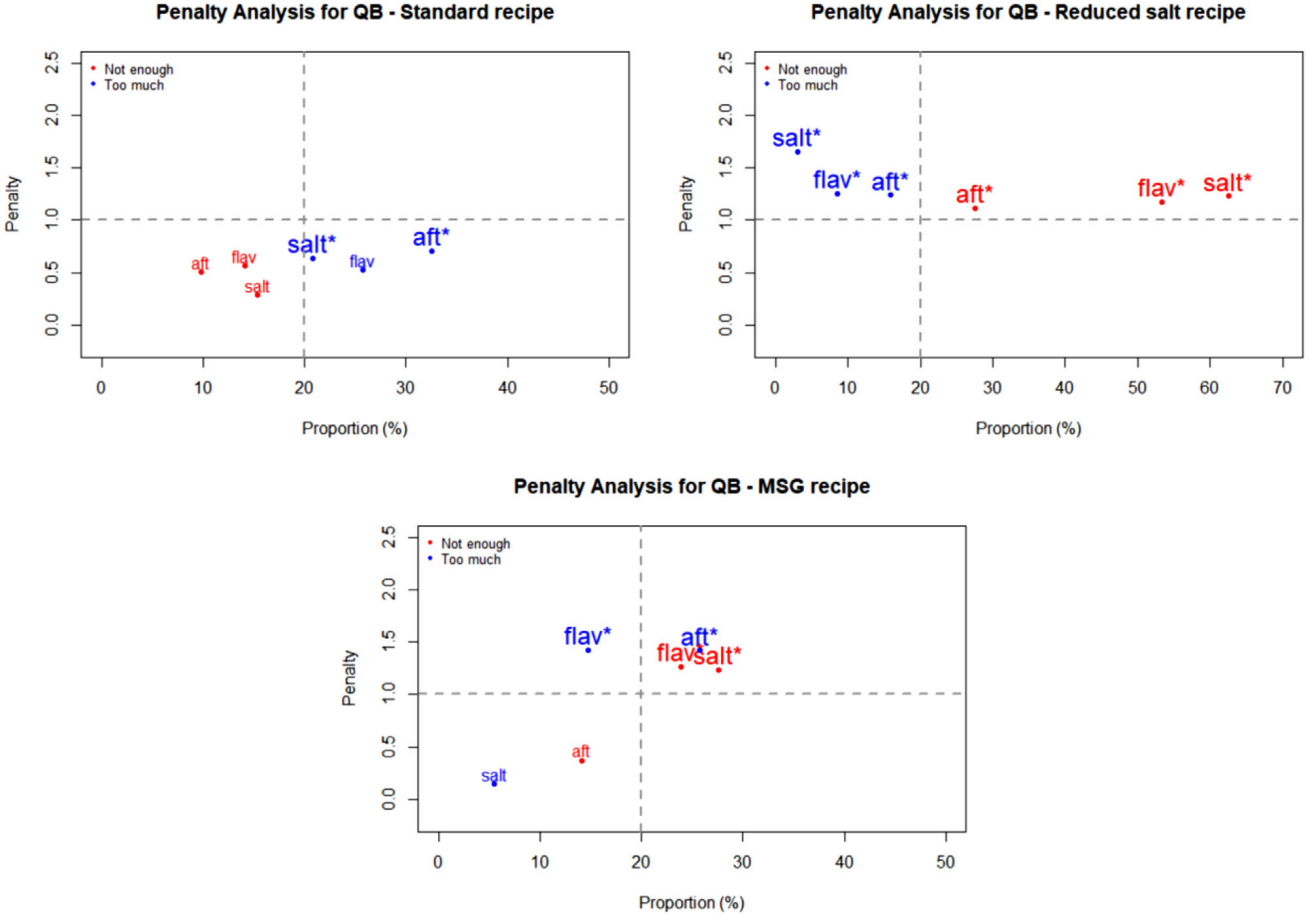
Penalty analyses of the three quinoa bowl (QB) recipes for three attributes: saltiness (“salt”), flavor (“flav”), and aftertaste (“aft”). *Note*: significant non‐JAR categories (*P* < 0.05) are shown in larger font size and marked with “^*^.”

**Figure 3:**
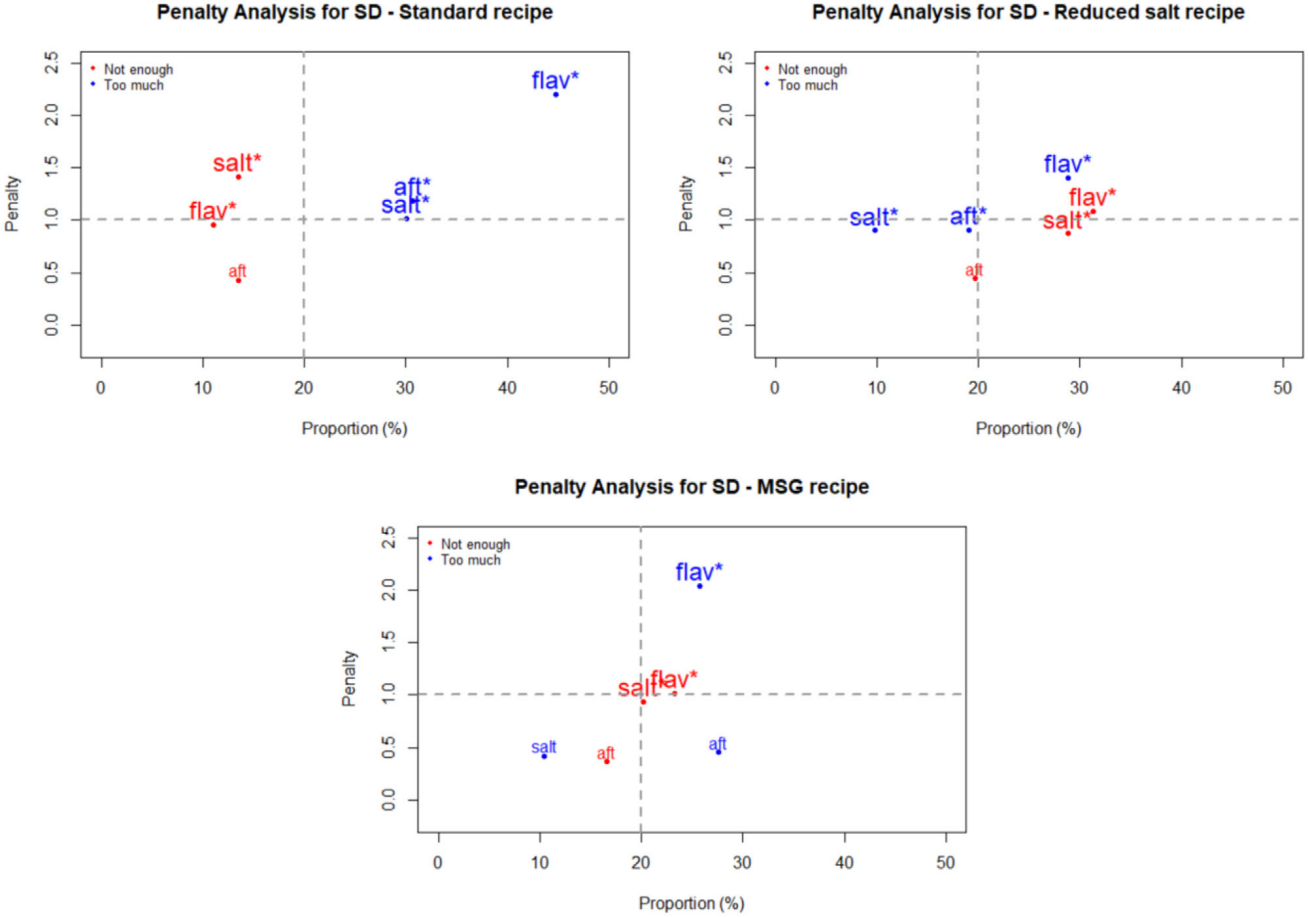
Penalty analyses of the three savory dip (SD) recipes for three attributes: saltiness (“salt”), flavor (“flav”), and aftertaste (“aft”). *Note*: significant non‐JAR categories (*P* < 0.05) are shown in larger font size and marked with “^*^.”

**Figure 4:**
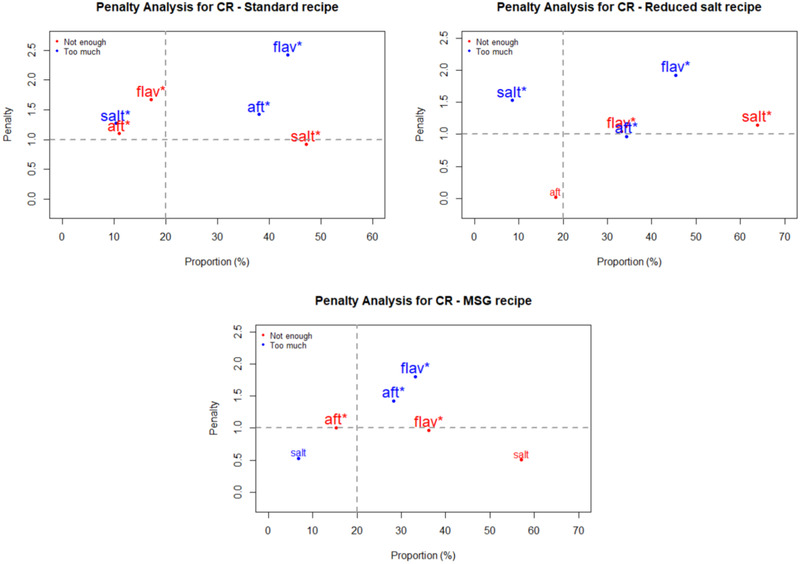
Penalty analyses of the three pork cauliflower rice (CR) recipes for three attributes: saltiness (“salt”), flavor (“flav”), and aftertaste (“aft”). *Note*: Nonsignificant JAR categories (*P* < 0.05) are shown in larger font size and marked with “^*^.”

Penalty analyses were performed on the JAR data to relate the impact of an attribute being rated as too low or too high on overall liking of the recipes by consumers. The outcomes are plotted in Figures 1, 2, 3, 4 as mean drops in overall liking against the percentage of consumer selecting too low or too high. A proportion of 20% was used as the minimum for inclusion in the figures. Of special interest are attributes located in the upper right quadrant of the plots, because they represent a high percentage of consumers and large penalties. Moreover, significant non‐JAR attributes (*P* < 0.05) are highlighted with an asterisk (^*^) and printed with a larger font size on those figures (1, 2, 3, 4).

Figure 1 shows the penalty analyses for the three RV recipes. They received significant penalties (about two points on the nine‐point hedonic scale) when flavor was deemed too much. Additionally, too much of an aftertaste in the three recipes caused a significant drop in overall liking. For QB (Figure 2), the reduced salt recipe suffered significant penalties from flavor, saltiness, and aftertaste being rated as too low. The lack of flavor and saltiness in particular were reported by high proportions of consumers. In the MSG version of QB, inadequate flavor intensity (too much for some consumers and too little for others) correlated to significant mean drops in overall liking. This so‐called equal bimodal data (ASTM MNL‐63), however can be disregarded as both ends corresponded to less than 50% consumers (Table 5). For the three SD recipes (Figure 3), too much flavor caused the greatest penalties. This was especially important for both the standard and MSG recipes, for which mean drops in overall liking exceeded 2 points on the nine‐point hedonic scale. Finally, the same trend was also seen for CR (Figure 4)—too much flavor resulted in significant penalties for the three recipes. In addition, significant mean drops were also associated with the samples having too much aftertaste.

### 3.6 Check‐All‐That‐Apply (CATA)

The frequencies of selection of the 16 CATA attributes for the recipes are shown in Supplement C. There were no differences in frequency of selection of the attributes among the RV recipes after adjusting for the family‐wise significant level (*P*_adj_ > 0.05). For QB, Cochran Q analyses with permutation test and Benjamini–Hochberg *P*‐value adjustment showed that the reduced salt version had lesser selections of the flavorful, salty, complex, delicious, balanced, savory, and aftertaste attributes (*P* < 0.001), and greater selection of the bland one (*P* < 0.001), compared to the other two recipes. For SD, the MSG recipe was identified more often as sweet (*P* = 0.0018) than the others. For CR, sour and rancid were picked significantly less often for the MSG recipe (*P* < 0.001), whereas the reduced salt recipe was described less often as flavorful or savory than the others (*P* < 0.001).

**Figure 5:**
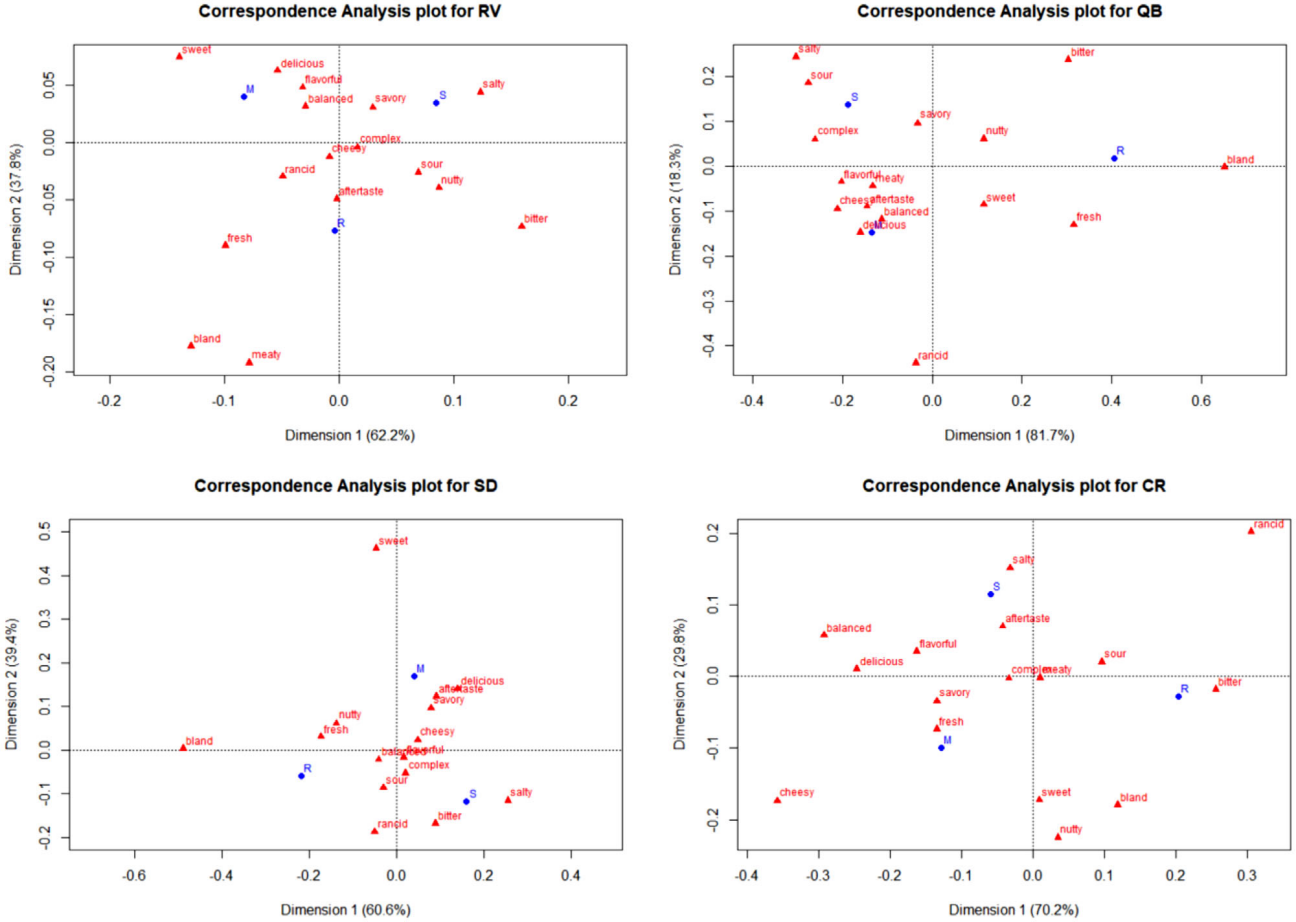
Correspondence analysis plots of CATA attributes for the three recipes (S: Standard, R: Reduced Salt, M: MSG recipe) of each dish (RV, QB, SD, and CR).

To visualize these relationships, symmetric biplots showing both the recipes and the CATA descriptors were constructed from correspondence analysis of the matrix of CATA selections across recipes for each dish (Figure 5). Generally, it can be seen that the reduced salt recipes of the four dishes were always associated with the attribute “bland.” They were also associated with the term “bitter” in both QB and CR dishes. The standard recipes were associated with the attribute “salty” in the four dishes. Interestingly, they were also associated with the term “sour” in both RV and QB dishes. The MSG recipes were positioned toward terms such as “delicious,” “flavorful,” and “balanced.” In addition, they were also associated with the term “savory” in the SD and CR dishes.

**Figure 6:**
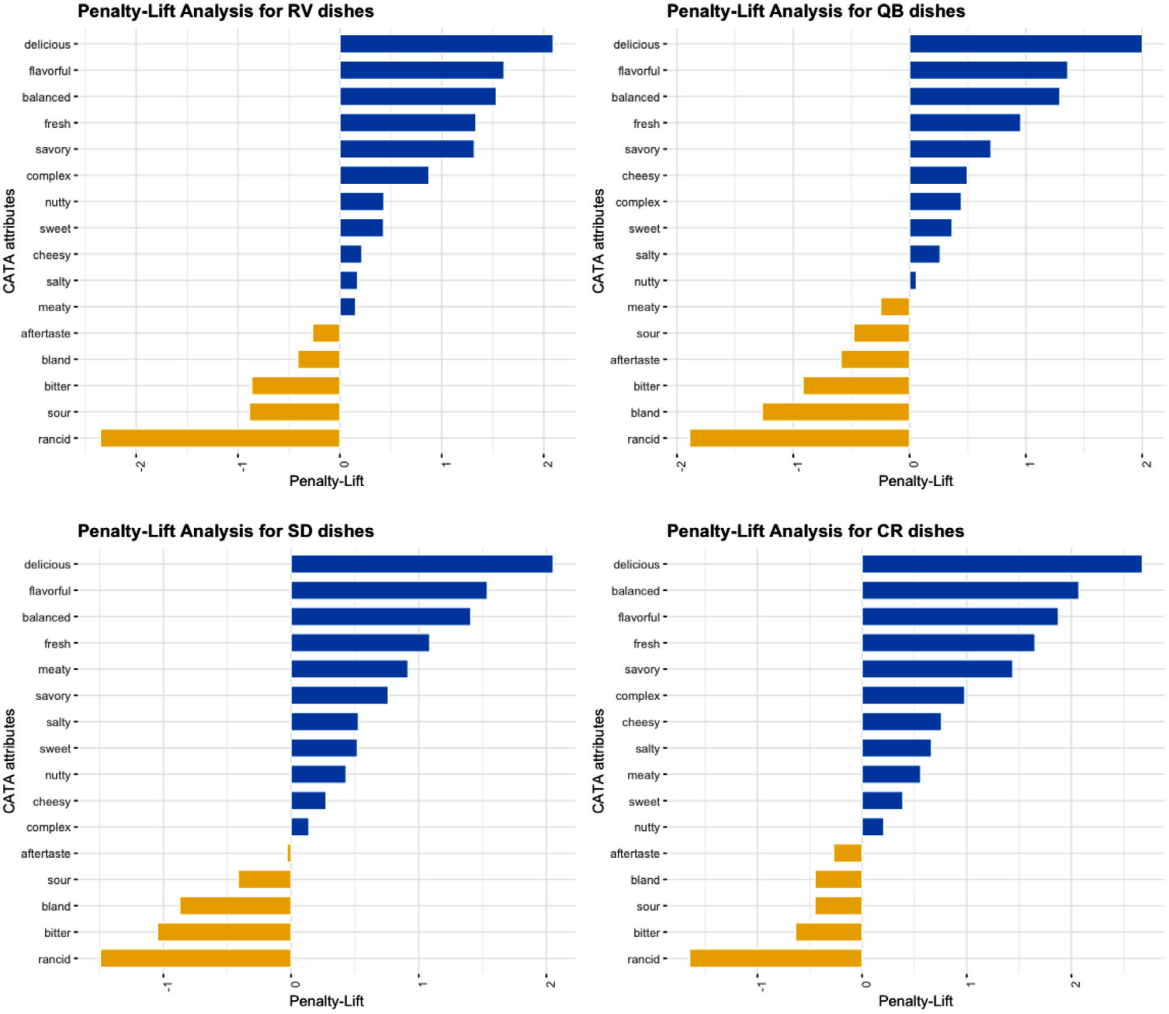
Penalty‐Lift Analyses relating CATA attribute selections to hedonic ratings for the four dishes.

Finally, the selection of CATA attributes was linked to the hedonic ratings for the recipes through penalty‐lift analysis (Figure 6). We found that for all dishes, the attributes delicious, flavorful, balanced, fresh, and savory consistently were positive drivers of liking (*P* < 0.05), whereas the attributes rancid, bitter, and bland consistently were negative ones (*P* < 0.05).

### 3.7 Preference mapping and clustering

**Figure 7:**
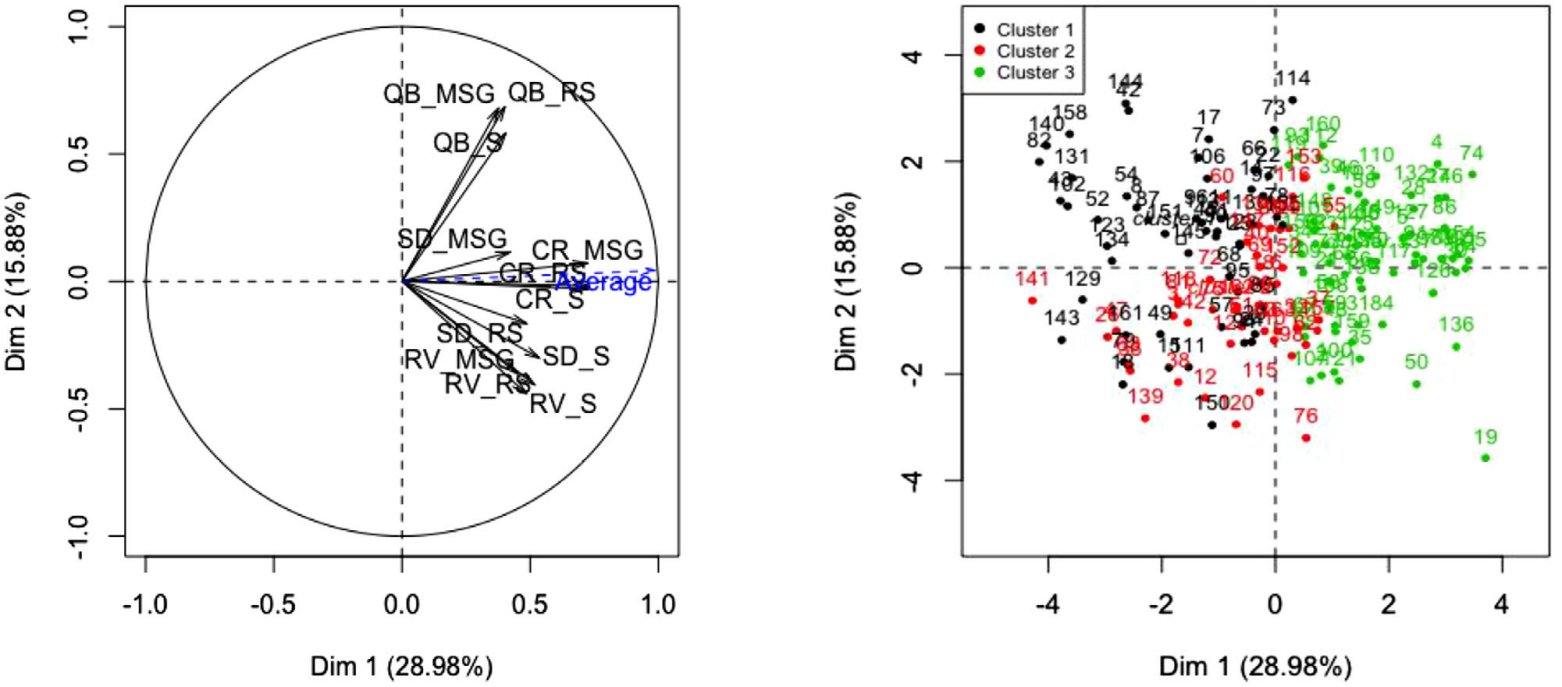
HCPC on uncentered overall liking matrix. Left: Factor map showing the PCA of the 12 samples and the vector of average overall liking. Right: Variable map showing the distribution of the three clusters of consumers on the first two principal components space.

HCPC of the overall liking ratings across the twelve (four dishes × three recipes) better‐for‐you foods in this study separated consumers into three preference clusters—cluster 1 (*n* = 52), cluster 2 (*n* = 43), and cluster 3 (*n* = 68; Figure 7). Consumers were loosely divided into those who liked everything (clusters 2 and 3) and those who were neutral or disliked everything along the first dimension (which accounted for 29% of the variance, a large amount given the dimensions of the data matrix). By contrast, differences in liking among the three versions within each dish were not as segmenting.

**Figure 8:**
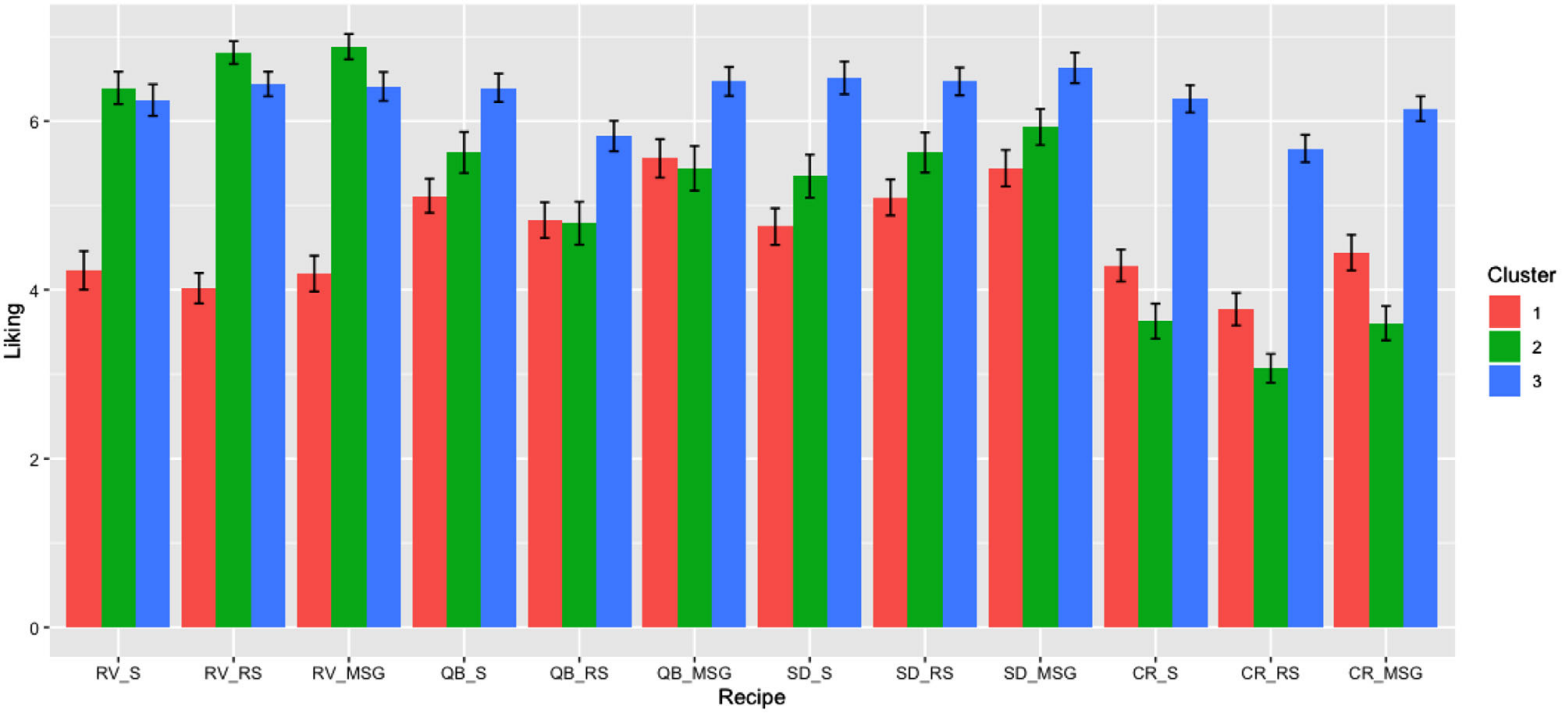
Average overall liking and mean standard error (SEM) by recipe for clusters 1 (*n* = 52), 2 (*n* = 43), and 3 (*n* = 68) based on HCPC of raw hedonic ratings.

It is potentially misleading to represent consumer acceptance as an average liking vector (shown in blue in Figure 7) when preference segmentation exists. So we examined the respective mean overall liking ratings of the three preference clusters for the various dishes and recipes (Figure 8). It is apparent that cluster 1, with 52 consumers, did not particularly like the (BFY) foods they tested, with mean hedonic ratings ranging between 4 and 6 on the nine‐point hedonic scale. But they liked (or disliked) the standard recipe and the MSG recipe the same for the RV and CR dishes, and actually liked the MSG recipe best for the QB and SD dishes. The 43 consumers in cluster 2 on the other hand, preferred the MSG recipe over the standard one for the RV and SD dishes, and showed parity in liking between them for the QB and CR dishes. Cluster 3, the largest with 68 consumers, liked the MSG and the standard recipes across all dishes (with almost all mean ratings above 6 on the scale), and the same.

An examination of the characteristics of the consumers in each preference segment (Table 6), as measured through the exit survey shows that cluster 1 included more Asians and consumers who used MSG more often than the other two clusters, yet those differences were not quite significant. Cluster 2 had the most diverse ethnicity and was characterized by a significantly higher stated liking for the taste and flavor of MSG (*P* = 0.026). Cluster 3, which liked the recipes more than the other two clusters, had more women and more Caucasians, yet the differences were not quite significant.

**Table 6:** Characteristics of consumer preference clusters based on HCPC on overall liking data

	Cluster 1 (*n* = 52)	Cluster 2 (*n* = 43)	Cluster 3 (*n* = 68)
Liking characterization	Dislikes everything	Likes RV, dislikes CR and QB (“low salt” and MSG)	Likes everything
Female (%)^a^	48.08	30.88	70.59
White/Caucasian (%)	19.23	27.91	35.29
Asian (%)	65.38	41.86	42.64
Mixed (%)	3.85	16.28	13.23
^b^Follow healthy and balanced diet	4.79	4.49	4.50
^b^Diet low in sodium	4.29	3.84	4.21
^b^Healthiness of food does not impact food choice	3.00	3.72	3.25
^b^Like the taste and flavor of MSG^c^	4.56a	5.26b	4.56a
^b^Aware that MSG can be used to reduce sodium level	3.88	4.30	3.94
^d^Frequency of meal preparation at home	3.92	3.72	3.91
^d^Frequency of using MSG^a^	1.77	1.46	1.37

Note: For gender and ethnicities, values represent percentages of the sample population. Percentage data were analyzed using Chi‐square analysis on the raw nominal data. For questions regarding eating habits and attitudes, values represent means of the raw data within clusters. The means were compared using nonparametric Kruskal–Wallis statistics.

^a^ Almost significant at *P* < 0.10.

^b^ Statements were evaluated on a seven‐point bipolar agreement scale with words, from “Strongly disagree” to “Strongly agree.”

^c^ Significant at *P* < 0.05, different superscript indicates differences using Duncan multiple comparisons.

^d^ Statements were evaluated on a 5‐point frequency categories: “Never or almost never” (1), “1‐3 times/month” (2), “1‐5 times/week” (3), “Daily or almost daily” (4), “Twice or more per day” (5).

### 3.8 MSG for mitigation of salt (and sodium) reduction

Our findings support the hypothesis that MSG can be added to plant‐forward, better‐for‐you dishes in which salt has been substantially reduced to maintain (or even improve) consumer acceptance and still achieve significant sodium reduction. Furthermore, all the measures of consumer acceptance we collected consistently support this hypothesis—overall degree of liking (both as average liking and liking by the preference segments we uncovered), liking of flavor, preference rankings, flavor, saltiness and aftertaste JAR ratings and likeliness to order (Tables 2, 4, 5 and Figures 7, 8). The sensory map that was derived from correspondence analysis of the CATA selections also serves to explain why that is, as all MSG recipes were characterized as “savory,” “flavorful,” “delicious,” and “balanced”—all holistic and/or hedonic descriptors with positive connotations in the context of these dishes (Figure 5).

What the preference mapping and clustering analysis showed is that the addition of MSG to the reduced‐salt recipes led to parity in (or even higher) liking with the standard recipes for a majority of the consumers in this study. We can make this statement because for any given dish, those preference segments with consumers rating the MSG recipe the same or higher than the standard recipe added up to more consumers than those with consumers who did not (Figure 8).

Our findings are therefore consistent with previous research that showed successful mitigation of salt reduction with MSG in a range of foods (Altug & Demirag, 1993; Chun et al., 2014; Jinap et al., 2016).

It is also worth noting that very little MSG was required to accomplish parity of consumer acceptance between standard and reduced‐salt recipes of four “better‐for‐you foods.” The sodium increase from the reduced‐salt recipe to the reduced salt with MSG recipe ranged from only 2% to 15% depending on the recipe (Table 1).

The FDA standard for a “reduced‐sodium” product is at least 25% less sodium than the original product. In this study, the application of MSG was successful in reducing sodium level by more than 30% from the standard recipes. This highlights the potential of MSG application in the formulation of “reduced‐sodium” foods. Moreover, for the CR dish, the MSG application reduced sodium by as much as 60%. This meets the FDA requirements for “light in sodium” labeling. We should also mention that pork, one of the main ingredients in the CR dish, contains 9 mg free glutamate and approximately 200 mg of other ribonucleotides per 100 g (Ninomiya, 1998), thus potentially working synergistically with MSG to boost flavor. This suggests that the extent of MSG application should consider the natural occurrence of glutamic acid and other naturally occurring flavor enhancing principles.

### 3.9 Cutting salt alone is not viable

The main challenge of reducing sodium content in foods may be the negative impact on the flavor profile and consumer acceptance (Hoppu et al., 2017; Liem, Miremadi, & Keast, 2011). This was confirmed in this study, as the reduced‐salt recipes received mostly lower ratings across most of the four dishes and most of the acceptance measures we collected. The fact that salt reduction only lowered overall liking for two of the four dishes (quinoa bowl and pork cauliflower fried rice), however, came as a surprise because the salt reduction (of 48% to 63%, depending on the dish) was substantial and comparable across dishes. Liem et al. (2011) indicate that a sodium reduction of up to 30% may be acceptable in processed foods if introduced gradually (over 3 years), and that the reduction can be up to 50% as long as it is in parallel with the addition of a flavor‐boosting ingredient, such as soy sauce or dried bonito.

And interestingly, the quinoa bowl and the pork cauliflower fried rice again were the only two dishes in which the hypothesis that MSG addition to the reduced‐salt recipe would increase liking was verified. It could be that the greater complexity of these two dishes, compared to the roasted vegetables or the savory yogurt dip, made for a better platform to illustrate the sensory effects of combined salt reduction and MSG addition. Indeed, all three hypotheses in this research were verified with these two dishes.

### 3.10 Hedonic scaling, preference ranking, and likeliness to order produce consistent results

As expected, preference rankings were consistent with the hedonic ratings, but they were also more discriminating, with a significant preference probability found by R‐index analysis for the recipes with MSG over the standard recipes in both QB and SD, whereas overall liking was only significantly higher in the case of SD; and a significant preference probability for the recipes with MSG over the reduced‐salt recipes for QB, SD, and CR, whereas overall liking was only significantly higher in the case of QB and CR (Tables 2 and 3).

Given that the main variables in this study were salt reduction and flavor enhancement with MSG, it was logical that the highest correlator to overall liking was liking of flavor.

Because the concept of Better‐for‐You foods is equally applicable to homes, takeout or food service and restaurants, we wanted to explore another measure of consumer acceptance in “likeliness to order.” We found some use of the measure in the literature (Anzman‐Frasca et al., 2014; McCall & Lynn, 2008). In their study of the effects of menu item descriptions on perception of quality, price, and purchase intention, McCall and Lynn (2008) found a highly significant correlation between perceived quality of the dish and likelihood of purchasing the item. Similarly, we found a highly significant correlation between hedonic ratings and likeliness to order. One would indeed expect a consumer to be likely to order a dish that he or she liked. There could also have been a halo effect from one measure (degree of liking) appearing first on the scorecard to another (likeliness to order) appearing after.

### 3.11 Flavor, saltiness, and aftertaste JAR scaling and CATA selections document how MSG works

The saltiness JAR ratings serve to validate our experimental design with regard to salt reduction. The incidence of just‐about‐right saltiness ratings were lower, and that of too‐low saltiness higher for the reduced salt recipes in all four dishes, especially for the QB dish even though the salt reduction was the lowest in that dish (Tables 1 and 5). Those findings were mirrored by the flavor JAR ratings, with too‐low flavor percentages going up for all four reduced salt recipes (again, most notably for the QB dish), thus emphasizing the key role of sodium chloride in flavor strength adequacy. And then, saltiness adequacy and flavor adequacy improved with the addition of MSG to the reduced salt recipes, with more just‐right saltiness ratings than reduced salt recipes for all dishes except CR, and more just‐right flavor ratings across all four dishes. These findings are all consistent with MSG boosting flavor and mitigating salt (and saltiness) reduction.

We included aftertaste in our JAR evaluations because it is a typical sensory characteristic of MSG and other umami tastants, and an overly lingering aftertaste can become an undesirable feature (Burseg, Camacho, & Bult, 2012; Giovanni & Guinard, 2001; Horio & Kawamura, 1990; Yamaguchi ad Ninomiya, 2000). But that did not seem to be the case here, as the MSG recipes did not receive significantly more too‐much aftertaste ratings than the standard, regular‐salt recipes (Table 5).

### 3.12 Consumers and MSG

Of all the consumers who participated in the study, the exit surveys revealed that only 16% considered themselves regular users of MSG. Of the remaining nonusers, 54% admitted that either they didn't know how to use MSG or they were not aware of it. About 23% of the non‐users did not use MSG due to availability issues. Finally, about 47% of the non‐users thought that MSG was not good for their health or were told not to use it by household members. It is unfortunate to see that the main reasons for not using MSG continue to be mainly awareness issues, either of its applications or health connotations. Despite a widespread belief that MSG can be detrimental for health (e.g. trigger for asthma or migraine headache), there is no consistent clinical evidence to support this claim. In fact, the safety of MSG has been evaluated by scientific committees and regulatory agencies and MSG is deemed to be safe. For comprehensive reviews on the safety aspects of MSG, see Freeman (2006), Jinap and Hajeb (2010), and Maluly, Arisseto‐Bragotto, and Reyes (2017). This research did not purport to examine consumer attitudes regarding MSG but rather focused on the sensory effectiveness and appeal of MSG in the mitigation of sodium reduction in a salt flip. Yet it is worth reiterating that attitudes regarding MSG have improved and that the outlook for its application in foods is positive. As stated in a recent Mintel report (2019), “Americans have been historically somewhat squeamish about seafood sauces (e.g., fish sauce, oyster sauce) and MSG for different reasons, but both of these items offer appealing umami taste benefits that consumers have only just begun to embrace with the help of celebrity chefs and restaurants. Look for fast future growth of these ingredients on restaurant menus in the coming years.”

### 3.13 Study limitations

Several ratings point to significant issues with the formulation of the pork cauliflower fried rice dish. In this study, however, we purposely used a simple baseline seasoning to be able to showcase the effects of salt and MSG more clearly. Furthermore, the recipes were plated without garnish to avoid possible halo effects from the presentation of the food, and that might have brought the hedonic ratings for the recipes down.

Finally, it should be noted that this study could have included many more versions of these prototypes with varying levels of salt and MSG to optimize results. Some optimization method, like response surface methodologies have been used to predict the optimum combination of MSG and salt in the past (Chi & Chen, 1992; Yamaguchi & Takahashi, 1984). However, this study provides a starting point for these prototypes of better‐for‐you foods.

## 4 CONCLUSION

We conclude that MSG can successfully be used in a Salt Flip to mitigate salt and sodium reduction without compromising consumer acceptance of better‐for‐you foods, particularly in complex dishes such as a quinoa bowl or pork cauliflower fried rice. This conclusion is supported across all the measures of consumer acceptance we collected in this study—overall liking (on average or by preference segment), liking of appearance, flavor and texture, preference ranking, likeliness to order and just‐about‐right scaling of flavor, saltiness, and aftertaste.

We can also document that for the better‐for‐you dishes we tested, the MSG recipes were consistently described as “savory,” “flavorful,” “delicious,” and “balanced” by consumers, thus pointing to the desirable way in which the parity in consumer acceptance between standard recipes and reduced‐salt with MSG recipes was achieved, and confirming MSG's already well‐documented flavor‐boosting properties.

## AUTHOR CONTRIBUTIONS

Author Halim collected and analyzed the data, and co‐drafted the manuscript. Authors Bouzari and Felder designed and prepared the recipes. Author Guinard designed the research and co‐drafted the manuscript.

## Supporting information


**Table A1**.RV dishes formulae; weight of ingredients (g) per 110 g.
**Table A2**.QB dishes formulae; weight of ingredients (g) per 140 g.
**Table A3**.SD dishes formulae; weight of ingredients (g) per 28 g.
**Table A4**.CR dishes formulae; weight of ingredients (g) per 119 g.
