# Supplementary material for: The Salt Flip: Sensory mitigation of salt (and sodium) reduction with monosodium glutamate (MSG) in “Better‐for‐You” foods
Source: J Food Sci. 2020 Aug 10;85(9):2902–14. doi: 10.1111/1750-3841.15354 (PMC7540316; doi:10.1111/1750-3841.15354)
Supplement: Supplementary file 1 — Table A1.RV dishes formulae; weight of ingredients (g) per 110 g. Table A2.QB dishes formulae; weight of ingredients (g) per 140 g. Table A3.SD dishes formulae; weight of ingredients (g) per 28 g. Table A4.CR dishes formulae; weight of ingredients (g) per 119 g. [file JFDS-85-2902-s001.docx]

**SUPPLEMENT A**

Formulae and Preparation of the Dishes

The formulae for the three recipes of each dish are shown in Tables A1 to A4, respectively for RV, QB, SD and CR dishes. The preparation steps needed were detailed for every section.

**Table A1**

RV dishes formulae; weight of ingredients (g) per 110 g

| **Ingredients** | **Standard** | **Reduced salt** | **MSG** |
| --- | --- | --- | --- |
| Carrots, raw | 42.45 | 42.58 | 42.45 |
| Eggplant, raw | 29.72 | 29.80 | 29.72 |
| Labne, full fat | 15.92 | 15.97 | 15.92 |
| Feta, greek | 10.51 | 10.54 | 10.51 |
| Oil, olive, extra virgin | 3.82 | 3.83 | 3.82 |
| Vinegar, sherry | 3.18 | 3.19 | 3.18 |
| Scallion, raw, minced | 2.55 | 2.55 | 2.55 |
| Parsley, raw | 0.58 | 0.59 | 0.58 |
| Cilantro, raw | 0.58 | 0.59 | 0.58 |
| Salt, kosher | 0.69 | 0.36 | 0.36 |
| Monosodium glutamate | - | - | 0.33 |

Preparation step:

1. Preheat oven to 425^o^F. Dice carrots and eggplant, and season with salt (and MSG), and a small amount of olive oil. Toss to coat.
2. Spread out Carrots, and Eggplant on separate sheet trays. Roast for 45 minutes, stir, and return to oven for 30-45 minutes or until nicely browned.
3. In a food processor, combine feta, parsley, cilantro, scallion, sherry vinegar, and olive oil. Process to a pesto-like texture. Reserve.
4. To serve, combine roasted vegetables, and feta mixture, and toss to combine. Spoon onto plate and top with vegetable mixture

**Table A2**

QB dishes formulae; weight of ingredients (g) per 140 g

| **Ingredients** | **Standard** | **Reduced salt** | **MSG** |
| --- | --- | --- | --- |
| Onion, cooked, white, minced | 73.30 | 73.54 | 73.32 |
| Quinoa, cooked | 56.97 | 57.15 | 56.97 |
| Oil, grapeseed | 8.79 | 8.81 | 8.78 |
| Salt, kosher | 0.94 | 0.50 | 0.50 |
| Monosodium glutamate | - | - | 0.43 |

Preparation step:

1. Combine quinoa, water (equivalent to 2x mass of raw quinoa), salt (and MSG). Bring to a boil, cover with a tight-fitting lid, reduce heat to par cook, simmering 10-15 minutes to achieve al-dente texture. Allow quinoa to rest, covered, for an additional 10-15 minutes before combining with cooked onions.
2. Separately, combine oil and minced onion, saute on medium heat for 20-25 minutes until translucent and golden brown.
3. Combine onion and quinoa mixtures, fold together to incorporate homogeneously.
4. Chill in sealed bags and store in refrigeration.
5. To serve, reheat sealed bags of quinoa mixture to minimum 150^o^F.

**Table A3**

SD dishes formulae; weight of ingredients (g) per 28 g

| **Ingredients** | **Standard** | **Reduced salt** | **MSG** |
| --- | --- | --- | --- |
| Yogurt, greek 5% fat | 25.37 | 25.58 | 25.45 |
| Oil, olive, extra virgin | 1.25 | 1.28 | 1.27 |
| Lemon, juice | 0.50 | 0.51 | 0.51 |
| Onion, powdered | 0.39 | 0.38 | 0.38 |
| Black pepper, ground | 0.08 | 0.08 | 0.08 |
| Salt, kosher | 0.41 | 0.17 | 0.17 |
| Monosodium glutamate | - | - | 0.14 |

Preparation steps:

1. Combine all ingredients and blend to incorporate homogeneously
2. Refrigerate until serving
3. Serve with 5 pc of saltine crackers as a supplement

**Table A4**

CR dishes formulae; weight of ingredients (g) per 119 g

| **Ingredients** | **Standard** | **Reduced salt** | **MSG** |
| --- | --- | --- | --- |
| Cauliflower rice, frozen | 46.42 | 46.60 | 46.58 |
| Ground pork, raw | 23.28 | 23.30 | 23.29 |
| Onion, raw, white, minced | 11.64 | 11.65 | 11.64 |
| Peas, IQF | 9.31 | 9.32 | 9.32 |
| Carrot, raw, diced | 7.92 | 7.92 | 7.92 |
| Scallion, raw, minced | 7.45 | 7.46 | 7.45 |
| Oil, sesame, toasted | 4.66 | 4.66 | 4.66 |
| Vinegar, rice, unseasoned | 2.33 | 2.33 | 2.33 |
| Ginger, raw, minced | 1.86 | 1.86 | 1.86 |
| Garlic, raw, minced | 1.86 | 1.86 | 1.86 |
| Red Pepper, ground | 1.86 | 1.86 | 1.86 |
| Salt, kosher | 0.43 | 0.16 | 0.13 |
| Monosodium glutamate | - | - | 0.11 |

Preparation step:

1. Preheat skillet or wok over high heat. Brown ground pork while breaking up into small pieces. Remove from wok, leaving some fat in the wok, and drain pork on towel lined tray.
2. Return wok to burner on high. add: carrot, and red pepper, and brown while tossing until lightly browned and softened. Add garlic, ginger, and peas to the wok. Toss to combine and cook for 30 more seconds. Reserve on tray with pork.
3. Add sesame oil to the wok and return to burner. Add cauliflower rice and cook until lightly browned. Add pork and vegetable components and toss to combine. Season with rice wine vinegar, and salt (and MSG).
4. Chill for refrigerated storage and reheat to 150^o^F for serving.

**SUPPLEMENT B**

Exit Survey

| Panelist name: ___________________    A1. What is your gender? (circle one below)  Male Female Other Prefer not to answer    A2. What is your age (in years)? _________    A3. What is your ethnicity? (circle all that apply)  White/Caucasian Black/African Hispanic/Latino Asian  Native American Mixed Other:__________________  Prefer not to answer    A4. What is your annual household income? (circle one below)  Less than $25,000 $25,000 to $49,999 $50,000 to $99,999  $100,000 to $149,999 $150,000 to $249,999 $250,000 or more  Prefer not to answer    A5. What is the highest level of education you have completed? (circle one below)  Less than high school  High school graduate  Some college, no degree  Bachelor's degree  Graduate or professional degree    A6. Please rank how often you eat these cuisines, with rank 1 being the most often. You are allowed to give ties for the ranks.  ___ Western (American, European)  ___ Southeast Asian (Thai, Malaysian, Indonesian, Filipino, Singaporean, etc.)  ___ Chinese  ___ Middle Eastern  ___ Japanese  ___ Korean  ___ Indian  ___ Latin American  ___ Other: _________________ (please mention)    *Using the scales below, please indicate how you feel about the following statements*  B1. I always follow a healthy and balanced diet   \| Strongly disagree \| Disagree \| Somewhat disagree \| Neither agree nor disagree \| Somewhat agree \| Agree \| Strongly Agree \| \| --- \| --- \| --- \| --- \| --- \| --- \| --- \|   B2. It is important for me that my diet be low in sodium   \| Strongly disagree \| Disagree \| Somewhat disagree \| Neither agree nor disagree \| Somewhat agree \| Agree \| Strongly Agree \| \| --- \| --- \| --- \| --- \| --- \| --- \| --- \|   B3. The healthiness of food has little impact on my food choices   \| Strongly disagree \| Disagree \| Somewhat disagree \| Neither agree nor disagree \| Somewhat agree \| Agree \| Strongly Agree \| \| --- \| --- \| --- \| --- \| --- \| --- \| --- \|   B4. How often do prepare your own meals at home?   \| Never or almost never \| 1-3 times/ month \| 1-5 times/ week \| Daily or almost daily \| Twice or more per day \| \| --- \| --- \| --- \| --- \| --- \|     B5. How often do you use MSG (monosodium glutamate) to prepare your food?   \| Never or almost never \| 1-3 times/ month \| 1-5 times/ week \| Daily or almost daily \| Twice or more per day \| \| --- \| --- \| --- \| --- \| --- \|     B6. I like the taste and flavor of MSG   \| Strongly disagree \| Disagree \| Somewhat disagree \| Neither agree nor disagree \| Somewhat agree \| Agree \| Strongly Agree \| \| --- \| --- \| --- \| --- \| --- \| --- \| --- \|     B7. I am aware and well informed that MSG can be used in the place of some salt to reduce the sodium level   \| Strongly disagree \| Disagree \| Somewhat disagree \| Neither agree nor disagree \| Somewhat agree \| Agree \| Strongly Agree \| \| --- \| --- \| --- \| --- \| --- \| --- \| --- \|     B8. If you do not consider yourself a regular user of MSG, what are the reasons why you don’t use MSG more often? (Check all that apply)  ☐ I would consider myself a regular user of MSG  ☐ MSG is not available where I shop  ☐ I don’t like the flavor of MSG  ☐ I don’t think MSG is good for my health  ☐ I don’t know how to use MSG  ☐ Other members of my household don’t like or will not consume MSG  ☐ I seldom prepare my own food  ☐ Other: _______________________________________ |
| --- | --- | --- | --- | --- | --- | --- | --- | --- | --- | --- | --- | --- | --- | --- | --- | --- | --- | --- | --- | --- | --- | --- | --- | --- | --- | --- | --- | --- | --- | --- | --- | --- | --- | --- | --- | --- | --- | --- | --- | --- | --- | --- | --- | --- | --- |

**SUPPLEMENT C**

Selection Frequencies of the CATA Attributes

| Dish | Recipe | Flavorful | Salty | Sweet | Sour | Bitter | Complex | Bland | Delicious | Fresh | Rancid | Balanced | Savory | Aftertaste | Nutty | Cheesy | Meaty |
| --- | --- | --- | --- | --- | --- | --- | --- | --- | --- | --- | --- | --- | --- | --- | --- | --- | --- |
| RV | S | 82 | 60 | 38 | 85 | 14 | 52 | 13 | 34 | 23 | 19 | 55 | 83 | 51 | 37 | 104 | 5 |
|  | RS | 75 | 46 | 38 | 80 | 13 | 50 | 22 | 31 | 31 | 21 | 52 | 73 | 55 | 35 | 105 | 8 |
|  | MSG | 87 | 44 | 52 | 70 | 9 | 49 | 18 | 38 | 29 | 21 | 58 | 76 | 50 | 29 | 104 | 6 |
| QB | S | 82 | 61 | 49 | 3 | 26 | 37 | 27 | 36 | 11 | 3 | 47 | 97 | 48 | 60 | 10 | 10 |
|  | RS | 40 | 18 | 56 | 1 | 30 | 14 | 84 | 22 | 21 | 4 | 32 | 66 | 29 | 58 | 5 | 6 |
|  | MSG | 87 | 36 | 64 | 2 | 14 | 32 | 35 | 49 | 18 | 9 | 61 | 79 | 58 | 54 | 12 | 11 |
| SD | S | 63 | 71 | 6 | 126 | 20 | 27 | 16 | 30 | 36 | 18 | 24 | 50 | 42 | 9 | 61 | 0 |
|  | RS | 55 | 33 | 9 | 119 | 14 | 23 | 50 | 20 | 51 | 17 | 24 | 39 | 32 | 12 | 50 | 0 |
|  | MSG | 61 | 48 | 19 | 107 | 13 | 24 | 29 | 37 | 46 | 12 | 24 | 59 | 52 | 12 | 63 | 4 |
| CR | S | 49 | 41 | 10 | 131 | 18 | 34 | 41 | 22 | 20 | 27 | 27 | 54 | 40 | 5 | 2 | 76 |
|  | RS | 29 | 27 | 12 | 132 | 25 | 28 | 61 | 11 | 15 | 29 | 11 | 38 | 29 | 7 | 1 | 68 |
|  | MSG | 45 | 27 | 14 | 109 | 15 | 32 | 57 | 22 | 23 | 11 | 25 | 57 | 32 | 8 | 3 | 70 |
